# Topology-Optimized Micromixer Design with Enhanced Reverse Flow to Increase Mixing Efficiency

**DOI:** 10.3390/mi14081599

**Published:** 2023-08-14

**Authors:** Qiang Fu, Zenghao Liu, Shuaiqi Cao, Zhe Wang, Guojun Liu

**Affiliations:** College of Mechanical and Aerospace Engineering, Jilin University, Changchun 130025, China; azithrom@foxmail.com (Q.F.); zenghao21@jlu.edu.cn (Z.L.); caosq21@jlu.edu.cn (S.C.); wzhe21@jlu.edu.cn (Z.W.)

**Keywords:** topology optimization, reverse flow, micromixer, computational fluid dynamics

## Abstract

In this work, a serpentine mixing unit model based on topology optimization is proposed to enhance the reverse flow in both horizontal and vertical directions. The increase in reverse flow in both directions can enhance the chaotic advection phenomenon, leading to a rapid increase in the mixing index. The proposed mixing unit model is applied in a T-shaped micromixer to create a new micromixer design, named TOD. Numerical simulations of TOD are performed using Comsol Multiphysics software to analyze the characteristics of the liquid flow, mixing surface, and pressure drop. The simulation results confirm that TOD has an outstanding mixing performance. By widening the surface area of contact and enhancing the chaotic advection phenomenon, TOD shows an excellent mixing performance at both a high and low Reynolds number, making it a promising micromixer design. For Re > 5, the mixing indexes of TOD are all beyond 90%.

## 1. Introduction

As an experimental application form of many disciplines such as chemistry and biology, microfluidic chips are receiving more and more attention [1,2,3]. Microfluidic chip technology can integrate the basic environment of discipline experiments into a chip of only a few square centimeters. The micromixer is a crucial component of microfluidic chips and plays a pivotal role in fluid mixing.

Based on the mixing mechanism, micromixers can be divided into two categories: active micromixers and passive micromixers [4]. The active micromixers rely on external energy sources such as acoustic waves, magnetic fields, and electric fields to disturb the internal fluid [5,6]. Passive micromixers do not require additional energy sources and rely on changes of channel geometry to alter the liquid trajectory, break up stratified flow, and achieve efficient mixing. Compared with active micromixers, passive micromixers have the advantage of lower environment requirements and lower manufacturing costs, making them increasingly popular in the field of microfluidics [7,8].

In recent years, various passive micromixers have been proposed. Jienan Shen et al. [9] optimized the design of diamond-like barriers using the finite element method and neural networks, which resulted in a higher mixing efficiency than the standard T-shaped micromixer. Hossain et al. [10] designed a micromixer with nonequilibrium triple diamond-shaped channels and investigated the effect of the geometric angle and channel width on mixing efficiency. In the passive micromixer with the serpentine channel, described by Kristina J. Cook et al. [11], the fluid is continuously forced to change its flow direction from the outer area to the inner area while being subjected to anisotropic pressure gradients, which results in helical motion and improves mixing. The serpentine channel creates the conditions for Dean flow generation. Shakhawat et al. [12] analyzed the effect of the barrier shape on a Tesla micromixer, in which circular barriers have a good mixing performance under the conditions of a smaller pressure drop while enhancing chaotic advection. Feng et al. [13] proposed two types of passive micromixers based on “XH”- and “XO”-shaped mixing units in which the fluid is periodically split, squeezed, and rotated in the mixing units. These researchers propose various types of structural designs that effectively enhance the mixing efficiency and functionality of micromixers. However, in the design process of the micromixers, due to the intricate fluid motion within microchannels, a considerable amount of experimental and numerical simulation work is required to achieve the optimal structure that corresponds to the desired mixing mechanism. In order to improve design efficiency, some researchers have started to explore the application of the topology optimization method in micromixer design. The topology optimization method can directly modify the structure shape to obtain the desired mixing mechanism, thereby improving the performance of micromixers and reducing the workload in the design process. Chen et al. [14] used the topology optimization method to improve the mixing performance of zigzag micromixers. The reverse velocity at the center of the microchannel is chosen as the objective function for topology optimization. Guo et al. [15] propose a topology optimization method based on the Lagrange mapping method, which is suitable for immiscible fluids where mixing is completely dominated by convection.

This study proposes a mixing unit model based on topology optimization to enhance mixing performance in microfluidic systems. The vertical velocity component of the specified point at the exit and the horizontal velocity component of the specified point at the entrance in the serpentine design space are minimized by topology optimization to enhance the reverse flow in both the vertical and horizontal directions. During the optimization process, an idealized porous material is assumed to fill the design region, and the controlling equations of the permeability at each location are iteratively solved to obtain a solid material distribution model that allows the liquid to flow in the opposite direction at specific points. Reverse flow in microchannels can enhance mixing performance and reduce mixing time. The proposed unit model is applied to a T-shaped micromixer, resulting in a new micromixer named TOD (topology-optimized micromixer design). The finite element method is used to simulate the comprehensive performance of the TOD. Extensive numerical data demonstrate that TOD exhibits an excellent mixing performance over a wide range of Reynolds numbers (0.01–100).

## 2. Numerical Analysis and Topology Optimization Methodology

In general, the analytical equations for the velocity and mixing of incompressible fluid flow are as follows [16]:

(1) $$(u\cdot \nabla )u=-\frac{1}{\rho }\nabla p+\nabla^{2}\eta u$$

(2) ∇⋅u=0

(3) $$(u\cdot \nabla )C=D\nabla^{2}C$$

where u (m/s) represents the velocity, p (Pa) represents the pressure, η (Pa·s) represents the viscosity, ρ (kg/m^3^) represents the fluidic density, C represents the concentration, and D represents the diffusion coefficient.

The mixing index, which reacts the mixing performance of species, can be defined as follows [16]:

(4) $$M=1.0-\frac{1}{c_{\infty }}\sqrt{\frac{\sum_{i=1}^{N}{\left(c_{i}-c_{\infty }\right)}^{2}}{N}}$$

where N is the number of sampling points inside the cross-section at the outlet, $c_{i}$ is the volume concentration fraction of sampling points, and $c_{\infty }$ is the volume concentration fraction of the idealized homogeneous mixture. The mixing index ranges from 0 (0%, not mixing) to 1 (100%, fully mixed).

The topology optimization in this article is implemented using the topological optimization module in COMSOL software. For the solution of nonlinear problems involving multiple degrees of freedom in topology optimization procedures, the method of moving asymptotes (MMA) is appropriate. The fundamental concept of MMA is to partition the design space into small regions and optimize each region iteratively. It can approximate the objective function by gradually adjusting damping coefficients to obtain the optimal solution.

The local permeability as the target parameter for topology optimization can be expressed as follows [17]:

(5) $$\alpha (\gamma )=\alpha_{\min}+(\alpha_{\max}-\alpha_{\min})\frac{q(1-\gamma )}{q+\gamma }$$

where γ = 0 represents the solid material, γ = 1 represents the liquid domain, q is a real and positive parameter that adjusts the convexity of the interpolation function, $\alpha_{\min}$ is the minimum value, α represents the impermeability, and $\alpha_{\max}$ is the maximum value. For impermeable solid domains, $\alpha_{\max}$ = ∞. But for numerical convergence, $\alpha_{\max}$ = 0, and $\alpha_{\max}$ is generally given a finite value.

The Darcy number and Reynolds number are two important dimensionless numbers in a fluid analysis. The Darcy number can be used to reflect the permeability of the porous medium and the Reynolds number can be used to define the fluid’s kinematic state. They are defined as follows [18]:

(6) $$Da=\frac{\eta }{\alpha_{\max}\cdot L^{2}}$$

(7) Re=uLρη

where L represents the hydraulic diameter of the fluid flow, and η represents the dynamic viscosity of the fluid.

The fluid topology optimization problem with the minimization of the objective function can be represented as follows [19]:

(8) $$\phi (u,\gamma )=\int_{\Omega }A(u,\gamma )dr+\int_{\partial \Omega }B(u,\gamma )ds$$

where A and B are the coefficients of each design field. In this paper, the velocity at two discrete specific points in the fluid domain is taken to be minimized, so that *A* and *B* are defined as follows [20]:

(9) $$\begin{array}{l} A\equiv \sum_{k=1}^{N}A_{k}(u,\gamma )\delta (r-r_{k})\;\;\;in\;\Omega \\ B\equiv 0\;\;\;on\;\partial \Omega \end{array}$$

where N represents the total number of the design element, $A_{k}$ represents the coefficients of the element k, Ω represents the fluid domain segment, ∂Ω represents the boundary segment, and $r_{k}$ represents the selected discrete point.

The optimization model in this work can finally be defined as

(10) $$\begin{array}{l} \min\phi (u,\gamma )\\ subject\;to:\;\rho (u\cdot \nabla )u=-\nabla p+\nabla \cdot \eta (\;\nabla u+{\left(\nabla u\right)}^{T})-\alpha (\gamma )u,\;\;\;\;\;\;\;\;\;\;in\;\Omega \\ \;\;\;\;\;\;\;\;\;\;\;\;\;\;\;\;\;\;\;-\nabla \cdot u=0\;\;\;\;\;\;\;\;\;\;\;\;\;\;\;\;\;\;\;\;\;\;\;\;\;\;\;\;\;\;\;\;\;\;\;\;\;\;\;\;\;\;\;\;\;\;\;\;\;\;\;\;\;\;\;\;\;\;\;\;\;\;\;\;\;\;\;\;\;\;\;\;\;in\;\Omega \\ \;\;\;\;\;\;\;\;\;\;\;\;\;\;\;\;\;\;\;\;0\leq \gamma \leq 1\;\;\;\;\;\;\;\;\;\;\;\;\;\;\;\;\;\;\;\;\;\;\;\;\;\;\;\;\;\;\;\;\;\;\;\;\;\;\;\;\;\;\;\;\;\;\;\;\;\;\;\;\;\;\;\;\;\;\;\;\;\;\;\;\;\;\;\;\;\;\;\;\;\;\;in\;\Omega \;\\ \;\;\;\;\;\;\;\;\;\;\;\;\;\;\;\;\;\;\;\;p=0,\;u=0\; \end{array}$$

For the constrained boundary condition, the iterative process of the topology optimization is as follows [21]:

- A proposed value is provided for $\gamma^{n}$ (the material distribution at step n), and the Navier–Stokes equations are solved to obtain the velocity field.
- $\phi^{n}$ (the objective function at step n) of $\gamma^{n}$ is obtained by performing the sensitivity analysis.
- γ ($\gamma^{n+1}$) is updated using MMA, and $\phi^{n+1}$ is calculated.
- The error ($\left|\phi^{n+1}\right.\left.-\phi^{n}\right|$) is calculated to determine whether to continue the iteration.

During the topology optimization process, the objective is to minimize the vertical velocity component of the selected point at the outlet and the horizontal velocity component of the selected point at the inlet within the serpentine design space, while simultaneously maintaining the liquid flow velocity in all other directions. This optimization process can induce the back-flow phenomenon and promote the expansion of contact areas between the combined solutions, making it more efficient for improving mixing.

**Figure 1:**
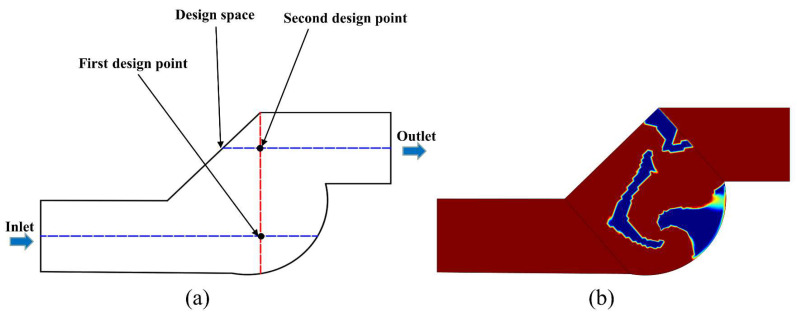
(**a**) Schematic diagram for topology optimization, blue dashed line the represents microchannel midline, red dashed line represents the unit cross-section; (**b**) results of the topology optimization, blue represents the solid material, red represents the liquid domain.

To achieve this, two design points are identified, as shown in Figure 1a. The first design point is located at the intersection of the entrance midline and the unit cross-section, and its −*u* value is chosen as an objective function to increase the horizontal reverse flow rate at the mixing unit’s entrance. The second design point is located at the intersection of the exit midline and the unit cross-section, and its −*v* value is chosen as the other objective function to increase the vertical reverse flow rate at the exit.

**Figure 2:**
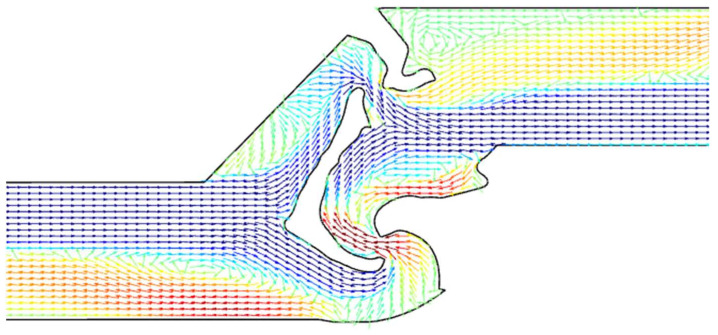
Velocity vectors in the topology optimization unit at Re = 100, blue represents the positive direction, red represents the opposite direction.

**Figure 3:**
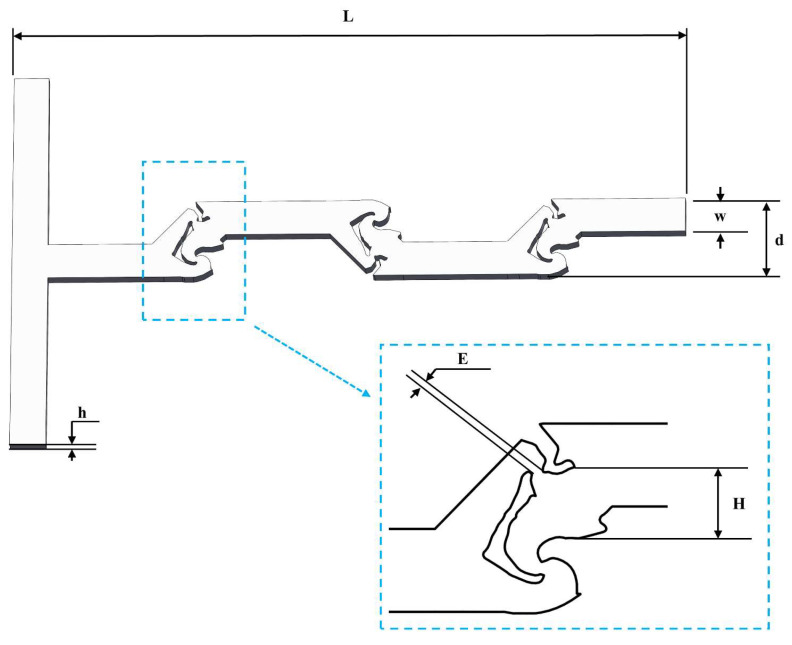
The micromixer based on topology optimization.

The result of the topology optimization is shown in Figure 1b, where the blue part indicates the solid domain and the red area indicates the liquid material. The transition zone with the sharp boundary lies between the two sections. If the topology-optimized geometry is directly adopted, sharp edges will result in manufacturing difficulties and high production costs. Therefore, before applying the topology-optimized geometry to the T-shaped micromixer, it is necessary to use image processing methods to smooth it. Figure 2 shows the velocity vectors within the optimized unit, where multiple flow reversals can be clearly observed both inside and outside the unit, leading to the different degrees of the vortex. Figure 3 shows the T-shaped micromixer structure with three topology-optimized mixing units that were smoothed, named TOD. The height of the microchannel is *h* = 1 mm, the width of microchannels is *w* = 2 mm, the total length of the TOD is *L* = 38.5 mm, the maximum width of the TOD is *d* = 4.5 mm, the minimum vertical distance between walls inside the mixing unit is *E* = 0.26 mm, and the maximum vertical distance between walls inside the mixing unit is *H* = 1.5 mm.

Comsol software is used to conduct a numerical analysis on the mixing process in the micromixer. The working fluid is assumed to be Newtonian and incompressible, with the dynamic viscosity of 26.5 × 10^−3^, the density of 1000 kg/m^3^, and the diffusion coefficient of 5 × 10^−10^ m^2^/s. The solute concentrations at the inlet of the two channels are 1 and 0 (mol/m^3^), respectively.

**Figure 4:**
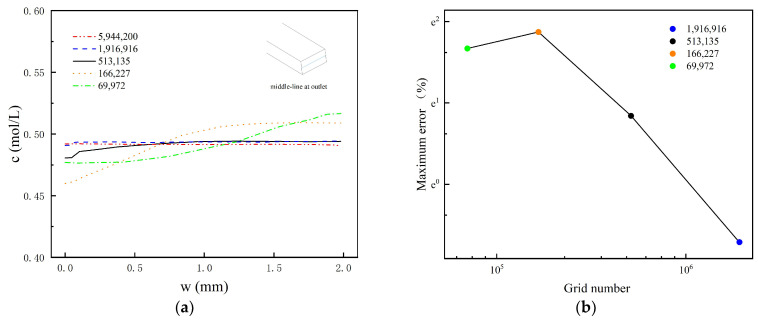
The grid independence test based on convection distribution. (**a**) The concentration distribution along the center line at the exit of TOD at Re = 100; (**b**) the maximum error (infinity norm) between different mesh curves and the curve with the 5,944,200 grid.

**Figure 5:**
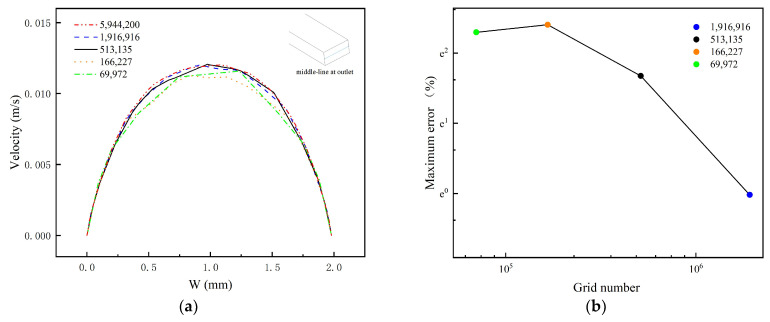
The grid independence test based on velocity distribution. (**a**) The velocity distribution along the center line at the exit of TOD at u = 0.003 m/s; (**b**) the maximum error (infinity norm) between different mesh curves and the curve with the 5,944,200 grid.

The accuracy of the simulation is affected by the quantity and distribution of the meshes. In this study, two grid independence test methods (the grid independence test based on the Navier–Stokes equation and the grid independence test based on the convection–diffusion equation) are used to determine the optimal meshing scheme of TOD. As shown in Figure 4, in terms of concentration distribution, the curves for the grid number of 1,916,916 and the grid number of 5,944,200 are very similar. The maximum error (infinity norm) between the two curves is no more than 0.5%. Likewise, Figure 5 shows that the velocity distribution curves for these two grid numbers are also very similar, with a maximum error (infinity norm) of no more than 1%. Therefore, the meshing method with the 1,916,916 grid is selected for numerical simulation, as they are sufficient to achieve a high level of simulation accuracy while also ensuring computational efficiency. This meshing system contains 1,635,317 tetrahedra, 142,890 triangles, 278,604 prisms, and 3380 pyramids.

## 3. Optimization Results and Discussion

### 3.1. Analysis of the Flow Characteristic

**Figure 6:**
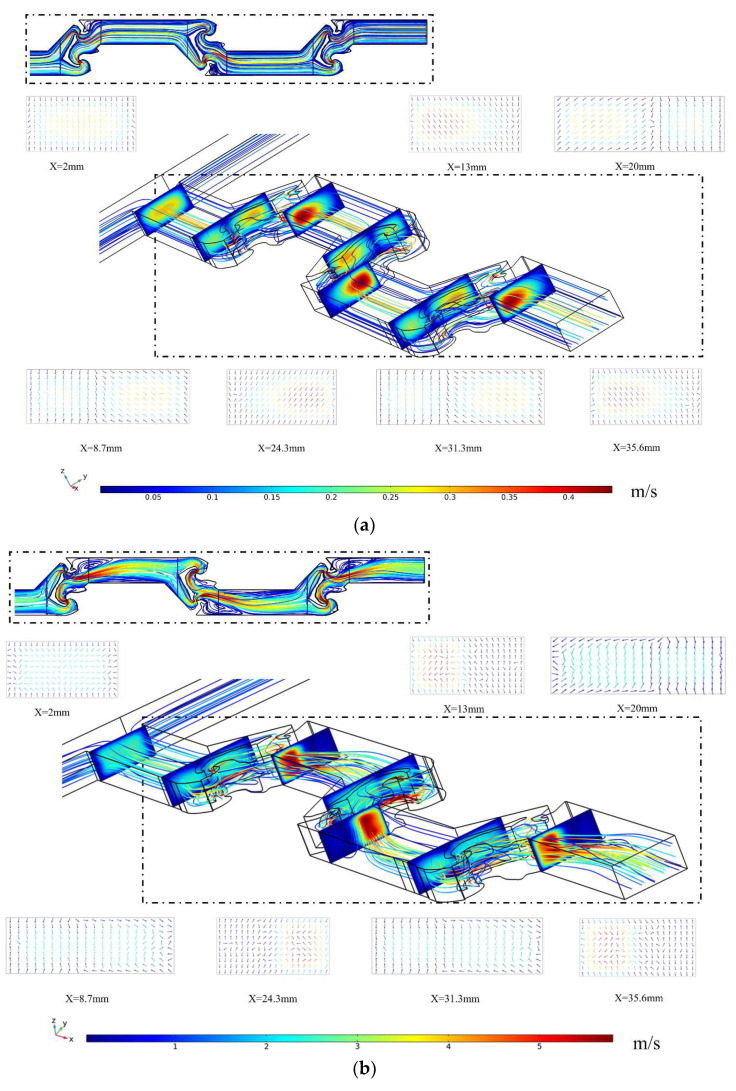

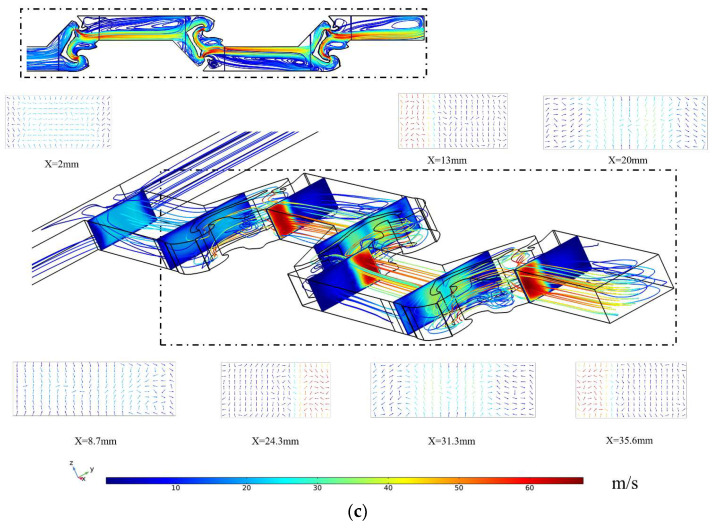
Flow characteristic of TOD at various Reynolds numbers. (**a**) TOD at the Re of 1; (**b**) TOD at the Re of 10; and (**c**) TOD at the Re of 100.

Figure 6 shows the flow characteristics and the vortex intensity of the representative cross-sections in TOD at different Re, where the colors represent the magnitude of the flow velocity and the direction of the arrow represents the main direction of the increasing vortex intensity. The fluid characteristics in the mixing unit can generally be described as follows: the fluids enter the main channel through the T-shaped inlet and collide to form a mainstream. This mainstream then reaches the first mixing unit, which includes topologically optimized baffles that separate the fluid into two sub-flows. The generation of sub-flows increases the contact area between solutions, and the sub-flows have varying velocities and flow rates due to the varying channel length and width. The two asymmetric sub-flows then intersect and collide, resulting in a vortex appearing on the low-velocity side area, further enhancing the mixing performance by increasing the interface area. Finally, the mainstream formed by the convergence of sub-flows leaves the mixing unit and flows through the convergence–divergence exit. Due to the quickly growing cross-sectional area at the exit of the mixing unit, Dean and expansion vortices are produced inside the microchannel. As shown in Figure 6a, when Re is 1, compared to the exit of the mixing unit, the velocity of the fluid is significantly reduced at the vicinity of the obstacles. It can be seen from the fluid’s streamline trajectory that the fluid enters a reverse flow region after passing through the obstacles. Under low Reynolds numbers (Re ≤ 1), molecular diffusion dominates the mixing process, and the mixing effect is governed by the flow residence time. The existence of topologically optimized obstacles and reverse flow regions increases the total path of fluid and prolongs the residence time, thus enhancing the mixing effect of the micromixer. Furthermore, by comparing the cross-sections and streamlines in Figure 6a–c, it can be seen that the intensity of vortices continuously increases with the increase in Re and more complex chaotic phenomena is generated in the reverse flow region. This indicates that under high Reynolds numbers, the obstacles’ base on topology optimization can increase the strength of chaotic convection and vortices, thereby improving the mixing performance.

### 3.2. Effect of Reynolds Number

**Figure 7:**
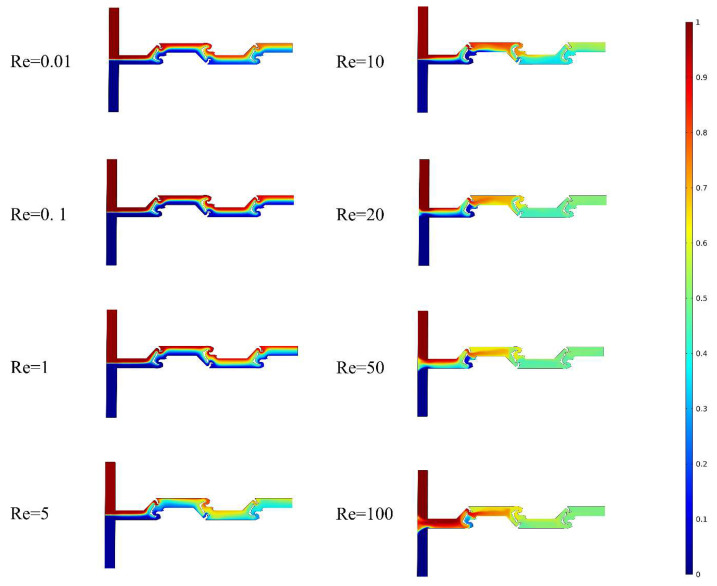
Concentration distribution in TOD at different Re.

The concentration surfaces within the micromixer at eight different Reynolds numbers are shown in Figure 7. When two fluids flow through the T-shaped inlet into the first mixing unit of TOD at different Reynolds numbers, the concentration color distribution of the two fluids is initially symmetric. However, as the fluid passes through the first mixing unit, a distinct transition color area appears on the concentration surface, which is closely related to the improvement in mixing performance in the microchannel. An increased range of the transition color area and a higher degree of color uniformity on the concentration surface are indicative of an improved mixing effectiveness. At low Reynolds numbers, the transition color area appears on the concentration surface of TOD, but there is no large area of a uniform color, and the range of the transition color area is small, indicating that the mixing effect inside the micromixer is not ideal. The reason for this is that under low Reynolds numbers, molecular diffusion dominates the mixing process. Although the mixing unit based on topological optimization increases the contact area between the solutions, the mixing process is slow due to the slow diffusion rate of molecules. Nevertheless, as the Reynolds number increases, chaotic convection gradually takes over the mixing process, leading to a rapid improvement in mixing performance. During this period, the range of the uniform color area on the concentration surface swiftly expands and moves towards the inlet, indicating a significant enhancement in mixing. When the Reynolds number reaches 10, the mixing is almost completely uniform. In summary, the topological design of the mixing unit effectively enhances chaotic convection and molecular diffusion. It is worth noting that even at low Reynolds numbers, TOD exhibits a good mixing performance, as evidenced by the rapid increase in the range of the transition color area and the color becoming more uniform as the Reynolds number increases from 1 to 5.

### 3.3. Mixing Comparison and Analysis

**Figure 8:**
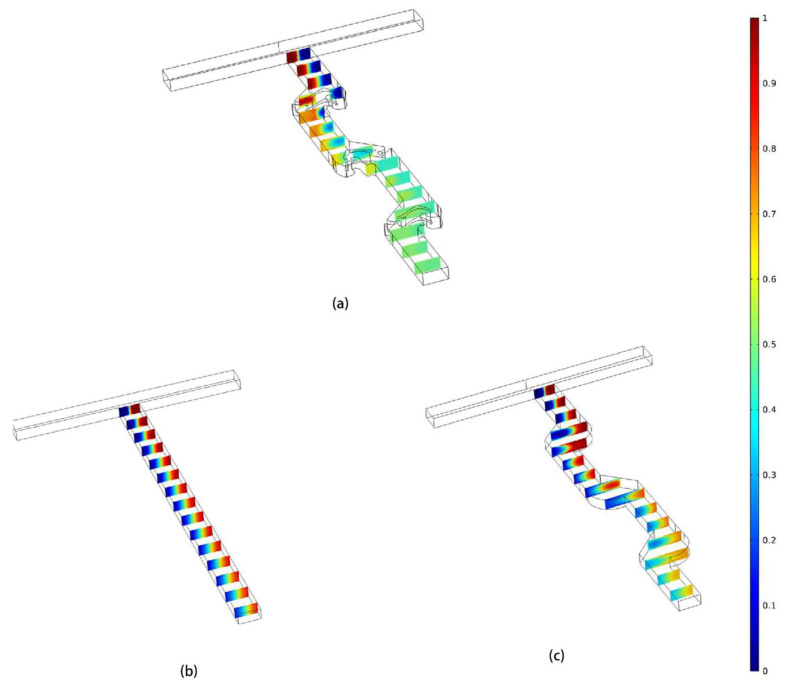
The concentration cross-sections along the microchannel at Re = 1. (**a**) TOD. (**b**) T-shaped micromixer. (**c**) Non-optimized serpentine micromixer.

Figure 8 shows the concentration cross-sections of three different micromixers, which are the T-shaped micromixer, non-optimized serpentine micromixer, and TOD. Compared to the other micromixers, TOD achieves a faster convergence of the two dominant concentration colors towards consistency.

**Figure 9:**
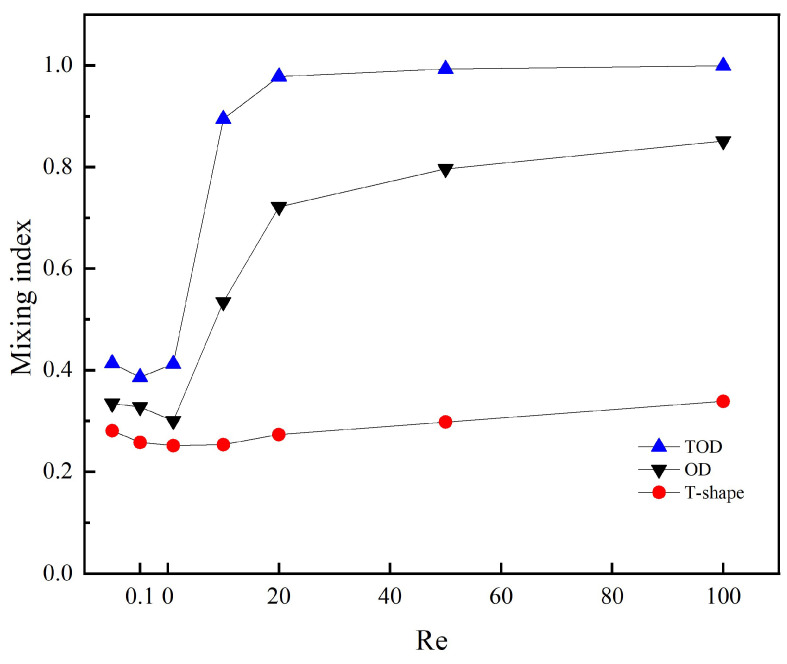
Mixing index of the three different micromixers.

To further analyze the mixing performance of the three micromixers, this study compared their mixing efficiency at the same axial length in the Reynolds number range of 0.01 to 100, as shown in Figure 9. Compared to the non-optimized serpentine micromixer and T-type micromixer, TOD shows a superior mixing performance throughout the entire Reynolds number range, particularly when the Reynolds number is greater than 1. Additionally, though the non-optimized serpentine micromixer shows similar mixing performance variation to the TOD when the Reynolds number is less than 1, the mixing index still has a difference of more than 10%.

### 3.4. Pressure Drop

**Figure 10:**
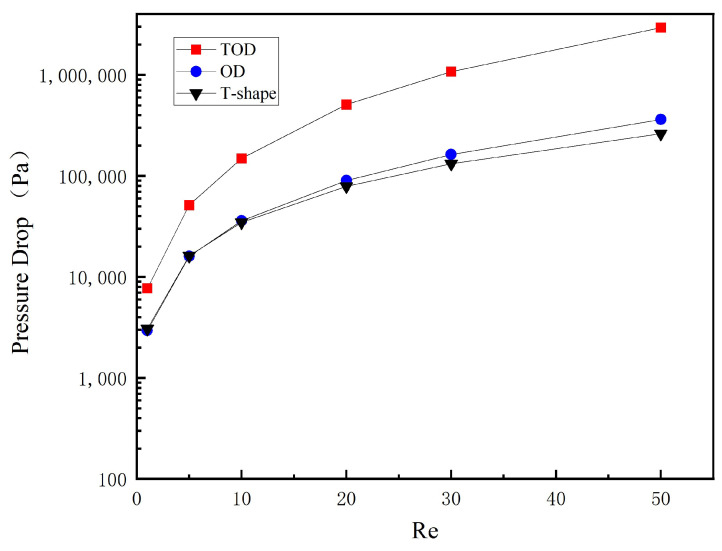
The pressure drop of the different designs at various Re.

A pressure drop is an important indicator for evaluating the comprehensive performance of micromixers, as it is closely related to the pumping power required. Figure 10 shows the pressure drop of three different micromixers at different Reynolds numbers. The pressure drop of the TOD at various Re is higher compared to the other two micromixers without topology optimization. Particularly under high Reynolds number conditions, the pressure drop is significantly increased by the generation of vortices and other complex flow phenomena. In fact, the high pressure drop is an unavoidable drawback of obstacle-based micromixers.

## 4. Conclusions

In this paper, a topology-optimized mixing unit model is proposed and applied to the T-shaped micromixer, named the TOD. Extensive numerical simulation data are used to analyze the mixing efficiency, pressure drop, and flow characteristics of the TOD. Based on the presented results, the major conclusions can be summarized as follows:

1. The topology-optimized mixing unit model is designed to generate reverse flow, which intensifies the secondary flow and, consequently, increases the contact area between solutions in the microchannel. This process leads to a significant enhancement in the mixing efficiency of TOD.
2. As the Reynolds number increases, the mixing index of TOD increases significantly, showing an excellent mixing performance in a wide range of Reynolds numbers, which is essential for chemical and biological engineering applications.
3. As the mixing effect improves, the pressure drop of TOD inevitably increases. A drawback of the obstructive micromixer is the high pressure drop. Nevertheless, except for some special application scenarios, TOD has promising application prospects in chemistry and related fields due to its excellent mixing performance.

## Data Availability

No new data were created or analyzed in this study. Data sharing is not applicable to this article.
